# Preparing for and conducting the National Health and Morbidity Survey in Malaysia amid the COVID-19 pandemic: balancing risks and benefits to participants and society

**DOI:** 10.5365/wpsar.2021.12.3.842

**Published:** 2021-09-03

**Authors:** Zhuo Lin Chong, Noor Aliza Lodz, Mohd Hatta Abdul Mutalip, Yin Cheng Lim, Maznieda Mahjom, Noor Ani Ahmad

**Affiliations:** a Institute for Public Health, National Institutes of Health, Ministry of Health, Setia Alam, Malaysia.; b Institute for Medical Research, National Institutes of Health, Ministry of Health, Setia Alam, Malaysia.; c Department of Social and Preventive Medicine, Faculty of Medicine, University of Malaya, Kuala Lumpur, Malaysia.

## Abstract

**Problem:**

The novel coronavirus disease 2019 (COVID-19) pandemic adversely affected the preparation of Malaysia’s National Health and Morbidity Survey for 2020 because conducting it would expose data collectors and participants to an increased risk of infection.

**Context:**

The survey is nationally representative and community based and is conducted by the Institute for Public Health, part of the National Institutes of Health, to generate health-related evidence and to support the Malaysian Ministry of Health in policy-making. Its planned scope for 2020 was the seroprevalence of communicable diseases such as hepatitis B and C.

**Action:**

Additional components were added to the survey to increase its usefulness, including COVID-19 seroprevalence and facial anthropometric studies to ensure respirator fit. The survey’s scale was reduced, and data collection was changed from including only face-to-face interviews to mainly self-administered and telephone interviews. The transmission risk to participants was reduced by screening data collectors before the survey and fortnightly thereafter, using standard droplet and contact precautions, ensuring proper training and monitoring of data collectors, and implementing other administrative infection prevention measures.

**Outcome:**

Data were collected from 7 August to 11 October 2020, with 5957 participants recruited. Only 4 out of 12 components of the survey were conducted via face-to-face interview. No COVID-19 cases were reported among data collectors and participants. All participants were given their hepatitis and COVID-19 laboratory test results; 73 participants with hepatitis B and 14 with hepatitis C who had been previously undiagnosed were referred for further case management.

**Discussion:**

Preparing and conducting the National Health and Morbidity Survey during the COVID-19 pandemic required careful consideration of the risks and benefits, multiple infection prevention measures, strong leadership and strong stakeholder support to ensure there were no adverse events.

## Problem

Novel coronavirus disease 2019 (COVID-19) was first reported in December 2019 in Wuhan, China, and a pandemic was declared by mid-March 2020. (*1*) The pathogen that causes COVID-19, severe acute respiratory syndrome coronavirus 2 (SARS-CoV-2), was first detected in Malaysia in three imported cases on 24 January 2020. (*2*-*4*) In early 2020, Malaysia experienced two waves of COVID-19 that flattened by the beginning of June. Then in September, the third wave began, during which the number of daily reported cases rose from less than 40 to a peak of more than 5000 in late January 2021. (*5*) As of late May 2021, the fourth wave was ongoing, with daily reported cases beginning to exceed the third wave’s peak. (*5*)

The COVID-19 pandemic has affected not only the population’s health and economic activity in Malaysia, but also its regular community health research projects. Here, we describe how health researchers in Malaysia adopted changes while preparing for and conducting a national survey amid the pandemic to balance the risks and benefits to the participants and society.

## Context

In 2020, Malaysia had an estimated population of 32.7 million. This number is expected to continue growing in the coming years. (*6*) To protect and improve the health of the population, the Malaysian Ministry of Health (health ministry) must review its programmes and priorities, evaluate the impacts of its health strategies and make plans for future resource allocation. These activities require evidence from timely and nationally representative community-based health data. These data are collected yearly by the Institute for Public Health through the National Health and Morbidity Survey (NHMS), a cross-sectional community-based survey. Data collected previously included information about the burden of various diseases and their risk factors, health-related behaviours, health care demand, health expenditures and disability. (*7*)

The focus of the NHMS 2020 was on communicable diseases, primarily hepatitis B and C, to be assessed through seroprevalence studies that would quantify the proportions of the population with immunity and with active and chronic disease. Although Malaysia is moving towards the goal of eliminating viral hepatitis by 2030, there have been no nationwide, population-based estimates of the burdens of hepatitis B and C. (*8*, *9*) The NHMS 2020 was designed to provide robust estimates to strengthen the health ministry’s prevention and treatment programmes targeting viral hepatitis and to achieve the elimination target.

The initial plan was to centrally recruit and train 22 teams of 7 data collectors. The data collectors would be sent to 4400 homes in communities across Malaysia that had been randomly preselected. There, the data collectors would recruit up to 12 000 eligible participants and interview them in their homes. In addition, multiple meetings and workshops would be scheduled with relevant stakeholders before data collection to aid in planning and after data collection to aid in data analysis and report writing. (*7*)

The COVID-19 outbreak in Malaysia, and the subsequent Movement Control Order imposed by the government, created many uncertainties around preparing for and conducting the NHMS 2020, especially in areas such as procurement, hiring and logistics planning. The house-to-house data collection, planned to occur from June to August 2020, might confuse participants and reduce the response rate to the population census that would be conducted later that same year. The survey would also increase the risk of COVID-19 among participants and data collectors.

## Action

Because the NHMS 2020 faced potential cancellation or postponement, researchers at the Institute for Public Health aimed to increase the benefits of the survey as a whole and reduce the risks of COVID-19 transmission to participants and data collectors. Therefore, the trajectory of the COVID-19 pandemic internationally and locally was closely monitored while work continued to prepare for the survey. We also revised our target sample size, from 12 000 to 6000 participants, and reviewed literature on COVID-19 research and guidelines on its prevention and control. (*10*, *11*) At the same time, stakeholders from within the health ministry and other institutions were engaged in discussions and negotiations to adapt the survey to reflect the latest and anticipated developments of the pandemic.

By April 2020, COVID-19 was estimated to be underreported due to subclinical or asymptomatic infections that could contribute to silent transmission. A COVID-19 seroprevalence study was necessary to quantify the actual burden and inform prevention and control strategies. (*12*) Because the NHMS 2020 already included seroprevalence studies requiring blood draws by phlebotomists, a COVID-19 seroprevalence study was added. Adding a facial anthropometric study to develop a respirator fit test panel for Malaysia was also proposed because this is essential to provide adequate protection for the population. These and other proposed measures are summarized in **Box 1**.

Box 1 **Measures proposed for the preparation and conduct of the National Health and Morbidity Survey 2020 in Malaysia to increase its benefits and reduce the risk of COVID-19 transmission**

Measures undertaken before data collection included:

adding COVID-19 seroprevalence and facial anthropometric studies (to develop a respirator fit test panel);
reducing the level of statistical analysis (from subnational to national) and reducing the overall survey target sample size from 12 000 to 6000 participants;
shortening the duration of data collection, from 3 months to 2 months;
downsizing the data collection team from 22 teams to 12 teams, with 7 data collectors each, comprising one experienced field supervisor from the Institute for Public Health, 5 research assistants and 1 phlebotomist;
changing the data collection method from exclusively face-to-face interviews to mostly self-administered questionnaires and computer-assisted telephone interviews;
recruiting well trained and experienced health staff as phlebotomists;
ensuring pre-training or pre-deployment SARS-CoV-2 reverse transcription polymerase chain reaction (RT–PCR) screening for all trainers and data collectors;
limiting the number of trainers and data collectors attending in-person training, which included a session on COVID-19 prevention, with training for phlebotomists delivered virtually;
ensuring that attendees at in-person training practised physical distancing and hand hygiene, and wore a face mask as their minimum personal protective equipment (PPE);
printing standard operating procedures for all staff.

Measures undertaken during data collection included:

limiting initial visits to selected homes to only 10 minutes and only for the purpose of recruiting eligible participants and distributing self-administered questionnaires;
collecting data in a public place within a community or at the nearest health facility to simplify compliance with additional infection prevention measures, such as:
making appointments to avoid crowding among participants;
providing a face mask (if the participant was not wearing one) and hand sanitizer upon arrival;
screening participants for fever and symptoms upon arrival at the registration counter;
providing a separate entrance and exit, with participants moving through the area in only one direction;
ensuring physical distancing between participants and data collectors, except during blood draws;
providing PPE for data collectors that followed standard droplet precautions, including a single-use face mask, face shield and long-sleeve gown;
limiting interaction to 15 minutes at each station (registration, interview and blood draw);
ensuring that data collectors and phlebotomists implemented hand hygiene and surface sanitization after interactions with each participant;
ensuring that clinical waste was properly managed;
screening data collectors with SARS-CoV-2 RT–PCR or a rapid antigen test kit every 2 weeks during the survey;
ensuring that compliance was monitored by the field supervisor and audited by Institute for Public Health researchers during regular visits.

Apart from the survey’s benefits to society, the hepatitis B and C seroprevalence studies would detect undiagnosed cases and ensure that they were referred for further case management. Additionally, all participants would be informed of their test results, whether positive or negative. Lastly, each participant was compensated for their time and transport costs.

This research project was approved by the Medical Research Ethics Committee under the health ministry (approval numbers NMRR-19–867–47973, NMRR-20–1166–55133 and NMRR-20–1217–55489).

## Outcomes

COVID-19 seroprevalence and facial anthropometric studies were added to the NHMS 2020 to further justify the survey ethically and financially. Data were collected in face-to-face interactions only for these two components and the hepatitis B and C components (blood draw and interview). Self-administered questionnaires were used to collect information about risk factors for blood-borne infections, HIV-associated stigma, and cognitive, affective and behavioural risk factors for dengue and zoonoses; computer-assisted telephone interviews were used to collect data about tuberculosis-like symptoms, antibiotic use, HIV knowledge and malaria prevention strategies.

Field data collection lasted about 2 months, from 7 August to 11 October 2020. A total of 5957 participants were recruited into the study. Audits during the survey found that compliance with standard operating procedures (SOPs) for COVID-19 prevention was high. Almost all proposed measures were implemented fully. However, there were three deviations. First, two teams were added during the last month of data collection to ensure that the survey remained on schedule and reached the target sample size. Second, although all trainers and data collectors were screened for COVID-19 before training, we could not ensure that this first screening was specifically by reverse transcription polymerase chain reaction (RT–PCR) because some of the tests were performed locally. Also, a few phlebotomists were not screened before their deployment. Third, the fortnightly COVID-19 screening was performed slightly later than scheduled for half of the teams.

However, two important changes were made to the data collection plan to avoid areas where there was a high risk of COVID-19 transmission. Prior to the start of training and data collection in one state in East Malaysia, it entered a 14-day quarantine mandate due to rising COVID-19 cases. Thus, activities were reorganized and moved to the next state. Also, one of the communities selected for the survey had a COVID-19 outbreak while one of the teams was there. Although the households the team was recruiting were not affected, activities had to be terminated before the authorities imposed stricter lockdown measures.

Among the 226 people involved in the survey, from training to data collection and phlebotomy, 492 COVID-19 screening tests were performed and their results tracked. This included postdeployment screening, which was added, due to the increase in COVID-19 cases in some areas towards the end of data collection. Of these tests, 147 were RT–PCR and 287 were rapid antigen tests. The method used for 58 tests could not be determined. Nevertheless, all screening tests were negative. Also, participants who were contacted within 1 month of the computer-assisted telephone interviews reported no new COVID-19 cases.

A total of 73 cases of hepatitis B and 14 of hepatitis C were detected by the survey in participants who were previously undiagnosed and unaware of their infection. All were notified and referred for further case management. All participants received all their laboratory results.

## Discussion

The ethics, risks and benefits of any research project must be considered, even more so during a pandemic (**Box 2**). The NHMS 2020 was directly beneficial to its participants, by informing them about their COVID-19 and hepatitis status, and then referring them for further case management when necessary. The survey also benefited Malaysia overall by providing an evidence base for health policy-making. However, because the Hippocratic oath advises “do no harm,” no matter how beneficial surveys like the NHMS may be, the risks posed to participants must be minimized. During preparation for the NHMS 2020, we did not come across any literature that considered the ethical aspects of conducting population health research during the COVID-19 pandemic that provided adequately detailed steps to prevent disease transmission. One such article was published in July 2020, after planning had almost been completed. (*13*) While we could not use this article to plan for our survey, our ethical and practical considerations were similar to those the article presented.

Box 2 **Summary of the lessons learned**

Conducting a national health survey during a pandemic like COVID-19 requires more consideration of the risks and benefits it poses to participants and society.
Multiple measures are required to prevent COVID-19 transmission during a health survey. Their implementation should be closely monitored and audited.
Strong support from stakeholders, strong leadership, creativity and flexibility were instrumental to the success of this health survey conducted during the COVID-19 pandemic: there were no infections among the data collectors, phlebotomists and participants.

In the absence of directly relevant literature, we turned to other literature and guidelines, particularly the Malaysian national guidelines and World Health Organization recommendations, to aid in drafting our standard operating procedures for preventing COVID-19 transmission during the survey. (*10*, *11*) Because this novel disease was not adequately understood at that time, we took standard droplet and contact precautions, as well as additional measures, but did not fully rely on just one type of precaution (**Box 1****, ****2**). These precautions were shown to be crucial later when the SARS-CoV-2 rapid antigen test was found to be less sensitive in detecting asymptomatic COVID-19. (*14*, *15*) Thus, relying on only one measure, especially when test results were negative, could have led to a false sense of security and a disease outbreak. (*15*)

The Institute for Public Health received strong support to conduct the survey from all stakeholders, including policy-makers, researchers and research collaborators, and selected study participants (**Box 2**). It took the efforts of all parties directly involved in training and data collection to ensure that the SOPs would prevent COVID-19 transmission. However, because screening for all of the phlebotomists and four of the data collection teams was done locally, some phlebotomists were not screened, and not all had the more sensitive RT–PCR test for their first screening. Additionally, adherence to fortnightly screening was affected by each team’s location and schedule, as well as the availability of health facilities to perform screening. We believe that adherence to the SARS-CoV-2 screening protocol could be improved by effective communication, better coordination and closer monitoring and auditing.

Although we consider the absence of positive tests and COVID-19 cases associated with the NHMS 2020 to be the result of the well constructed and strictly followed SOPs, we recognize that the lower sensitivity of our screening methods and the lack of active COVID-19 cases among the communities we encountered may have also led to these good outcomes. We also relied on passive surveillance efforts in each community. Nevertheless, as our understanding of COVID-19 improves, we believe that our SOPs remain relevant and adequate.

Finally, we learned that planning and conducting a national health survey, such as the NHMS 2020, during a pandemic is not easy. Circumstances can change rapidly, even in the presence of advance planning. Strong leadership, creativity and flexibility were instrumental to this survey’s success (**Box 2**).

In conclusion, although our circumstances may differ from other researchers’, the principles we followed in preparing for and while conducting the NHMS 2020 during the COVID-19 pandemic can reduce the risks and increase the benefits of health research to participants and to society.
